# Effect of Repetition Rate on Femtosecond Laser-Induced Homogenous Microstructures

**DOI:** 10.3390/ma9121023

**Published:** 2016-12-19

**Authors:** Sanchari Biswas, Adya Karthikeyan, Anne-Marie Kietzig

**Affiliations:** Department of Chemical Engineering, McGill University, 3610 University Street, Montreal, QC H3A 0C5, Canada; sanchari.biswas@mail.mcgill.ca (S.B.); adya.karthikeyan@mail.mcgill.ca (A.K.)

**Keywords:** femtosecond laser, surface micromachining, microstructures, repetition rate, accumulated fluence, lacunarity analysis, copper, titanium

## Abstract

We report on the effect of repetition rate on the formation and surface texture of the laser induced homogenous microstructures. Different microstructures were micromachined on copper (Cu) and titanium (Ti) using femtosecond pulses at 1 and 10 kHz. We studied the effect of the repetition rate on structure formation by comparing the threshold accumulated pulse ($F_{\Sigma pulse}$) values and the effect on the surface texture through lacunarity analysis. Machining both metals at low $F_{\Sigma pulse}$ resulted in microstructures with higher lacunarity at 10 kHz compared to 1 kHz. On increasing $F_{\Sigma pulse}$, the microstructures showed higher lacunarity at 1 kHz. The effect of the repetition rate on the threshold $F_{\Sigma pulse}$ values were, however, considerably different on the two metals. With an increase in repetition rate, we observed a decrease in the threshold $F_{\Sigma pulse}$ on Cu, while on Ti we observed an increase. These differences were successfully allied to the respective material characteristics and the resulting melt dynamics. While machining Ti at 10 kHz, the melt layer induced by one laser pulse persists until the next pulse arrives, acting as a dielectric for the subsequent pulse, thereby increasing $F_{\Sigma pulse}$. However, on Cu, the melt layer quickly resolidifies and no such dielectric like phase is observed. Our study contributes to the current knowledge on the effect of the repetition rate as an irradiation parameter.

## 1. Introduction

Modification of the surface topography can substantially alter the properties of a surface. Over the years, direct laser micromachining, particularly with femtosecond (fs) lasers, has emerged as a novel and versatile surface modification technique due to certain advantages, such as its ability to micromachine all types of materials under any ambient condition without requiring clean room facilities or high vacuum equipment, and its ability to impart a large variety of hierarchical microstructures in a single processing step [1,2,3,4,5].

Microstructures are formed as a product of the various ablation mechanisms resulting from complex photo-physical processes triggered by the laser-material interaction. A number of factors such as electron-phonon (*e-ph*) coupling constant and thermal conductivity of the material, wavelength and polarization of the incident light, machining environment and other micromachining parameters, such as the fluence, determine the laser-material interaction and the following ablation mechanism. Since the nature of the micromachining outcome is strongly dependent on several factors, it is desirable to understand how these factors or parameters influence the microstructure formation. Consequently, considerable research effort has been made to study the effects of these micromachining parameters. While the effects of some parameters, such as the laser fluence, scanning velocity, number of scans, and polarization of the light are studied exhaustively [6,7,8,9,10,11,12], few reports exist that study the effect of the laser repetition rate as a machining parameter [10,13,14,15]. Yet again, most of these studies are on single spot experiments, reporting either the variation of ablation depth, ablation rate, or the threshold fluence with repetition rate. However, fabrication of the different microstructures, such as the bumpy or pillow-like structures, conical, and undulated groove-like structures, requires some sort of partial pulse overlapping [16] and to the best of our knowledge, no studies have been reported so far that provide any insights on the effect of repetition rate on laser induced microstructures formed as a result of partially overlapping pulses. For instance, a well-defined correlation between the threshold fluence required for the formation of the microstructures or the structural evolution of the microstructures with the repetition rate does not exist within the Hz and kHz range [14,17,18], which happens to be the range where most microstructures on metals were reported (see Table 1). Thus, it is worthwhile to investigate the effect of the repetition rate on the micromachining outcome to predict microstructure formation.

In this work, we report the effect of repetition rate on the micromachining outcome. Our group had previously reported on the machining of a number of microstructures on copper (Cu) and titanium (Ti) at 10 kHz and on the use of the accumulated fluence profile (AFP) irradiation model in mapping the evolution of the microstructures [23]. Following the same procedure and analysis, we micromachined Cu and Ti at 1 kHz and plotted the evolution of our fabricated microstructures. These evolution plots, generated at 1 kHz and 10 kHz, were then compared. This allowed us to directly analyze the effect of repetition rate on microstructure evolution. Additionally, we analyzed the lacunarity (surface texture) of the microstructures at the two repetition rates to identify any topological changes caused due to change in the repetition rate.

## 2. Materials and Methods

### 2.1. Materials

Cu (99.9% purity, McMaster-Carr) and Ti (Grade 2, 98.9% purity, McMaster-Carr, Elmhurst, IL, USA) were used in this study. Prior to laser micromachining, all samples were polished with 600 and 1200 grit sandpaper and ultrasonicated in acetone for 5 min.

### 2.2. Laser Micromachining

Samples were attached to a linear *x*–*z* translation stage (Newport Corporation, Irvine, CA, USA) controlled by the GOL3D software (GBC&S, Brie Comte Robert, France). A Ti:Sapphire laser (Coherent Libra, Santa Clara, CA, USA) with wavelength 800 nm, pulse duration <100 fs, and repetition rate (*f*) of 1 kHz was used for micromachining area patches. A 100-mm focusing lens was used to focus the horizontally polarized Gaussian laser beam onto the sample. The maximum output power of 4 W was reduced to the desired processing power with a variable attenuator composed of a half-wave plate and a polarizing beam splitter. At first, the effective line width ($\omega_{eff}$) was determined from the line scans obtained at a respective power (*P*), scanning velocity (*v*), and position of the sample from the focus (Δ*y*). Thereafter, the area patches (1 mm by 1 mm) were micromachined in a raster scan pattern. To achieve that, a single line was machined at first by overlapping the pulses in the horizontal (*x*-) direction in displacements of Δ*x* (=*v*/*f*). Thereafter, these horizontal lines were overlapped (overlap percentage, $\varphi_{line}$) in the vertical (*z*-) direction to machine an area patch. The machining parameters (*P*, *v*, Δ*y*, $\varphi_{line}$) were varied over a wide range and a number of raster scans were carried out at several possible combinations of these parameters to yield various microstructures. All samples were raster scanned once, and were ultrasonicated after micromachining in acetone for 5 min to remove the nanoparticle debris.

### 2.3. Structure Analysis

#### 2.3.1. SEM and Optical Microscopy

The $\omega_{eff}$ was measured by an optical microscope (Leica EZ4 D, Wetzlar, Germany). The resulting mico- and nano-structures were analyzed by scanning electron microscopy (SEM) (FEI Inspect F50, Hillsboro, OR, USA) and categorized into different types based on their structures.

#### 2.3.2. Lacunarity (*λ*)

The spatial heterogeneity of hierarchical microstructures can be conveniently studied by lacunarity analysis. The lacunarity plots can be utilized to highlight structural changes, mainly in terms of their coarseness or texture, caused by the changes in the machining parameters. In our experiments, any structural differences between the microstructures brought about by the change in repetition rate were identified from their lacunarity analysis, which was carried out using their SEM images.

Typically the lacunarity is calculated using the sliding-box counting algorithm [24], where a box of dimension *r* × *r* slides across a binary image of size *M* × *N* and the number of foreground (white) and background (black) pixels in that box is measured at each position along the image generating a distribution. The lacunarity *λ* of the image is then calculated from the distribution:

(1) $$\lambda \left(r\right)={\left(\frac{s_{k}\left(r\right)}{\bar{k}\left(r\right)}\right)}^{2}+1$$

where $s_{k}$ and $\bar{k}$ represent the standard deviation and mean of the foreground pixel distribution respectively at a given box size *r*. Equation (1) shows that λ(r) is a measure of the ratio of the standard deviation of the foreground pixels to their mean at a given *r*. As the image becomes more spatially heterogenous (greater variance), the λ(r) increases, alternatively suggesting that an image with a higher lacunarity value is spatially coarse or clumped, while an image with a lower lacunarity value corresponds to fine texture. The highest value of λ(r) is obtained at *r* = 1, where the lacunarity depends only on the proportion of the foreground pixels relative to the entire image and is equal to 1/*P*, where *P* is the total number of foreground pixels divided by the total number of pixels. When *r* approaches the image size *M*, the normalized standard deviation of the foreground pixels approaches zero and λ(r) tends to unity. However, for structure comparison, looking at lacunarity values at a single *r* does not reflect the overall texture, since λ(r) is dependent on the box size *r*. One way of generating a single lacunarity parameter is by calculating the area under the ln(*λ*) vs. ln(*r*) curve

(2) $$\theta =\overset{\ln M}{\underset{0}{\int }}\ln\left(\lambda \right)dln\left(r\right)$$

where *M* is the smaller dimension of the image being analyzed and θ is known as the spatial heterogeneity index. A detailed formulation of λ(r), its limiting cases, and the use of θ in analyzing the structural hierarchy can be found in the recent study by Ling et al. [25]. The lacunarity analysis presented in this study was carried out following the same procedure.

For each structure, three SEM images at 1000× magnification (1024 pixels × 842 pixels) were captured from surfaces of the same structure type but micromachined at different settings. At this magnification, 1 pixel represents ~0.29 µm. During image acquisition, the brightness and contrast of the SEM images were adjusted to ensure maximum consistency. The grayscale SEM images were converted to binary images by first equalizing their histograms and then applying a threshold of 0.5 to maintain constant foreground and background pixels. Thereafter, using the software plugin FracLac in ImageJ and the sliding-box counting method, the analysis was carried out for each microstructure. This resulted in plots of ln(*λ*) as a function of ln(*r*). The effect of changing repetition rate on the microstructures’ lacunarity was studied by comparing their *θ* values calculated from each lacunarity plot.

### 2.4. Accumulated Pulse Fluence (AFP) Model

Partial pulse overlapping is necessary to extend microstructure formation over an area [16]. Consequently, it is not justified to use the pulse fluence to analyze the microstructures. The accumulated fluence resulting from partially overlapping Gaussian pulses can be calculated using the AFP irradiation model [16,26]. The fluence distribution of individual pulses is given by:

(3) $$F_{p}\left(x,y,z\right)=\;\left(\frac{8P}{\pi \omega_{theo}^{2}f}\right)\exp\left(-\frac{8\left(x^{2}+z^{2}\right)}{\omega_{theo}^{2}}\right)$$

where $\omega_{theo}$ is the theoretical beam diameter. Summing up individual successive pulses in the horizontal displacement of Δ*x* gives the measure of the accumulated pulse fluence, $F_{\Sigma pulse}$, which results in a single line scan. Summing successive line scans in the vertical displacements of Δ*z* (= (1 − $\varphi_{line}$)$\omega_{eff}$) gives the measure of the accumulated line fluence, $F_{\Sigma line}$, which is equivalent to the resulting fluence deposited over the reference area. Use of the AFP model is advantageous not only because it gives a measure of the total fluence distribution over a reference area, but it also converges all the micromachining parameters like *P*, *v*, Δ*y*, and $\varphi_{line}$ into two parameters, the pulse—($F_{\Sigma pulse}$), and the line—($F_{\Sigma line}$) accumulated fluence. During comparison, it is straight forward to work with just these two parameters in contrast to considering all four micromachining parameters individually.

### 2.5. Repetition Rate Comparison

The effect of repetition rate on microstructure formation was studied by comparing the evolution plots of the microstructures machined at 1 kHz and 10 kHz generated using the AFP irradiation model. Only the homogenous microstructures were considered for comparison. At first, we identified the different types of homogenous microstructures from their SEM images and calculated their $F_{\Sigma pulse}$ and $F_{\Sigma line}$ values from their respective machining parameters. Thereafter, we plotted the microstructures as a function of $F_{\Sigma pulse}$ and $F_{\Sigma line}$. The threshold $F_{\Sigma pulse}$ values, at which a specific microstructure started to appear, were identified for each homogenous microstructure and the effect of the repetition rate was studied by comparing these threshold $F_{\Sigma pulse}$ values obtained at 1 and 10 kHz. It is worth mentioning that the fabricated microstructures are the result of pulse overlapping in displacements of both Δ*x* and $\varphi_{line}$. To assess the effect of changing repetition rate, $F_{\Sigma \mathrm{pulse}}$ values were considered which incorporate the repetition rate through Δ*x* directly into the accumulated fluence model. Thus, the comparison was based on the $F_{\Sigma pulse}$ values.

## 3. Results

### 3.1. Microstructures Fabricated at 1 kHz

#### 3.1.1. Copper (Cu)

**Figure 1:**
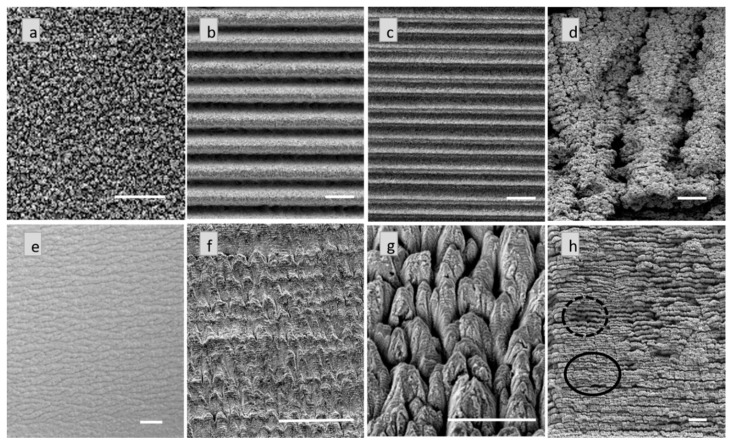
Representative images of the microstructures on Cu machined at 1 kHz (**a**) Nanoforest; (**b**) Deep and well defined trenches; (**c**) Narrow trenches; (**d**) Rough and rugged chaotic structures; (**e**) Tree bark; (**f**) Stalagmite structures; (**g**) Fish scales and (**h**) Aggregate structures showing the partial breaking (solid circle) of the top layer and the underlying structure (dashed circle). All scale bars represent 50 µm.

Micromachining Cu at 1 kHz yielded eight different types of microstructures. SEM images of the representative microstructures are shown in Figure 1a–h.

Out of these, the nanoforest like structures (Figure 1a), the deep and well defined trenches (Figure 1b), the narrow trenches (Figure 1c), and the chaotic microstructures (Figure 1d) were also reported on Cu micromachined at 10 kHz (Figure S1 in Supplemental Information) [23]. Since these four structures were reproducible on Cu at both 1 kHz and 10 kHz, they will be referred to as the common microstructures in later sections. Ripples were also produced at both 1 and 10 kHz, however, we will restrict our discussion purely to the microstructure formation as our interest lies primarily in studying the effect of repetition rate on more complex structures. Our experiments at 1 kHz yielded four additional microstructures; the tree bark structure (Figure 1e), the stalagmites (Figure 1f), the fish scales (Figure 1g), and the aggregate structures (Figure 1h). To the best of our knowledge, all four of these structures are reported for the first time. A comprehensive list of the micromachined structures is provided in Table 2.

While the tree bark structures consisted of interwoven long streaks of about 10 µm diameter running parallel to each other along the horizontal scan direction, the stalagmite structures consisted of saw-tooth like features of about 10 µm diameter directed towards the vertical scan direction. The fish scale structures were considerably larger than the rest of the microstructures having a diameter of about 25 µm and seemingly several layers stacked on top of one another. Interestingly, in the case of the aggregate microstructures, some regions were deeper than others (Figure 1h); such behavior was not observed for the rest of the microstructures. At first, this led us to believe that the top surface was just the nanoparticle debris that had partially broken off at the top layer. However, even with an increase in the sample cleaning time, we did not achieve complete removal of the top surface. Furthermore, the underlying structure did not resemble any other types of microstructures shown here. We recognized the observed morphology as another different class of microstructure, which we described as aggregate microstructures.

#### 3.1.2. Titanium (Ti)

**Figure 2:**
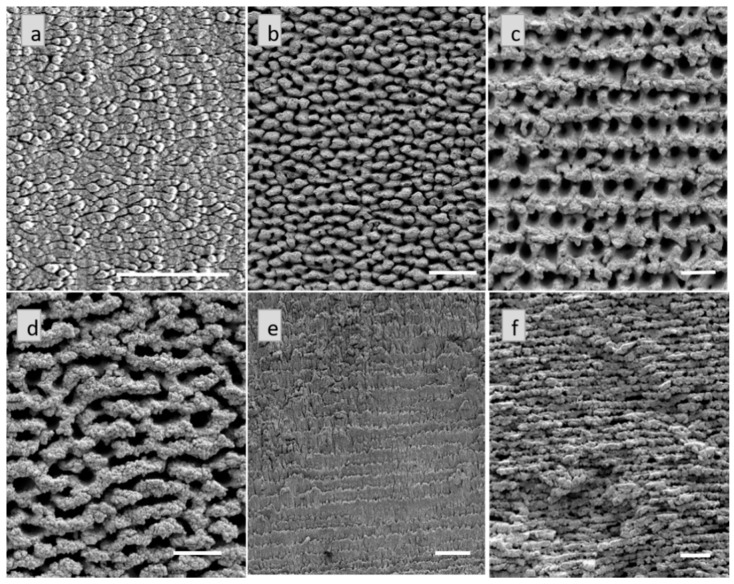
Representative images of the microstructures on Ti machined at 1 kHz. (**a**) Undulating grooves; (**b**) Bumpy structures; (**c**) Holes; (**d**) Chaotic structures; (**e**) Stalagmites and (**f**) Aggregate structures. All scale bars represent 50 µm.

Micromachining Ti at 1 kHz yielded six different types of microstructures. SEM images of the representative microstructures are shown in Figure 2a–f and are listed in Table 2.

Out of these, the undulating grooves (Figure 2a), the bumpy structures (Figure 2b), the holes (Figure 2c), and the chaotic structures (Figure 2d) were reproducible on Ti at both 1 and 10 kHz [23] (Figure S2 in Supplemental Information), and they will be referred to as the common microstructures on Ti. Additionally, micromachining Ti at 1 kHz, yielded two new structures; the stalagmite and the aggregate like structures. Again, to the best of our knowledge, here they are reported on Ti for the first time. Interestingly, the trench-like structures that were micromachined on Ti at 10 kHz [23] were not reproducible at 1 kHz. The stalagmite structures machined on Ti were noticeably different to the ones machined on Cu. Not only did they look smaller, they were less prominent on Ti as compared to Cu, where well-defined saw-tooth-like features existed. The aggregate structures on Ti, however, showed the same behavior of partial breaking at certain places, as seen on the Cu aggregate structures.

### 3.2. Effect of Repetition Rate

#### 3.2.1. Copper (Cu)

**Figure 3:**
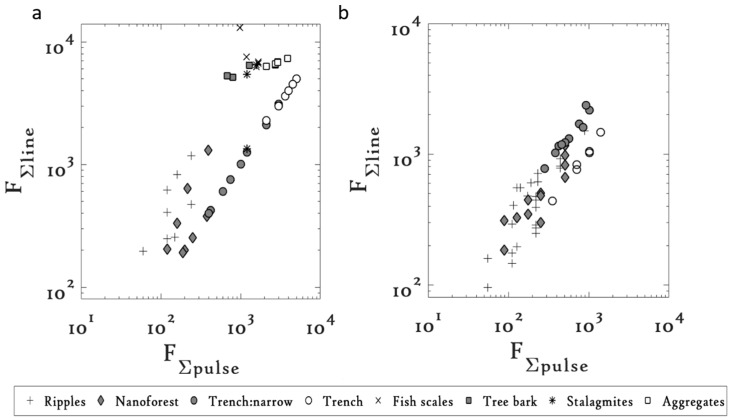
$F_{\Sigma line}$ vs. $F_{\Sigma pulse}$ plots on Cu at (**a**) 1 kHz and (**b**) 10 kHz [23]. (Reprinted from Optics and Lasers in Engineering, 66, K.M. Tanvir Ahmmed, Edwin Jee Yang Ling, Phillip Servio, Anne-Marie Kietzig, Introducing a new optimization tool for femtosecond laser-induced surface texturing on titanium, stainless steel, aluminum and copper, 258-268, 2014, with permission from Elsevier.)

The evolution of the microstructures on Cu machined at 1 and 10 kHz is shown in the $F_{\Sigma line}$ vs. $F_{\Sigma pulse}$ plots (Figure 3).

At both 1 kHz and 10 kHz, the common microstructure on Cu evolved from nanoforest like structures to form narrow trenches, which further gave way to form deep trenches with increasing $F_{\Sigma pulse}$. At 1 kHz, the nanoforest like structures required a threshold $F_{\Sigma pulse}$ of 120 J/cm^2^. Micromachining parameters that resulted in $F_{\Sigma pulse}$ values below this threshold generated ripples.

On increasing $F_{\Sigma pulse}$ above 400 J/cm^2^, the nanoforest like structures gave way to form narrow trenches. On further increasing $F_{\Sigma pulse}$ above 2101 J/cm^2^, the narrow trenches gave way to form deep trenches. Interestingly, when these threshold $F_{\Sigma pulse}$ values were compared to their counterparts obtained at 10 kHz, the threshold $F_{\Sigma pulse}$ were observed to have reduced for all the common microstructures (see Table 3).

For instance, the nanoforest structures that required a threshold $F_{\Sigma pulse}$ of 120 J/cm^2^ at 1 kHz required a threshold of 88 J/cm^2^ at 10 kHz. Similarly, for the narrow trenches that required a threshold of 400 J/cm^2^ at 1 kHz the threshold $F_{\Sigma pulse}$ decreased to 282 J/cm^2^ at 10 kHz. The deep trenches having a threshold of 2101 J/cm^2^ at 1 kHz, were fabricated at a much lower threshold $F_{\Sigma pulse}$ of 353 J/cm^2^ at 10 kHz. The persistent trend observed in all the common microstructures suggested a decrease in the threshold $F_{\Sigma pulse}$ values of the microstructures with an increase in the repetition rate.

#### 3.2.2. Titanium (Ti)

**Figure 4:**
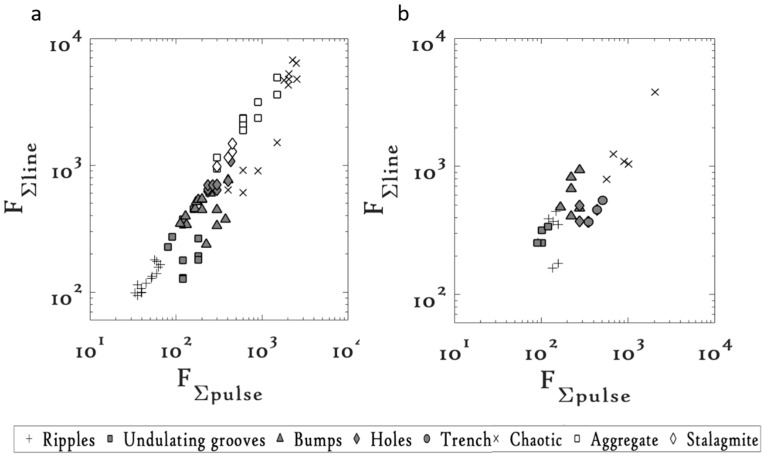
$F_{\Sigma line}$ vs. $F_{\Sigma pulse}$ plots on Ti at (**a**) 1 kHz and (**b**) 10 kHz [23].

The $F_{\Sigma line}$ vs. $F_{\Sigma pulse}$ plots for the common microstructures on Ti machined at 1 kHz and 10 kHz are shown in Figure 4 and their respective threshold $F_{\Sigma pulse}$ values are listed in Table 3.

With increasing $F_{\Sigma pulse}$, the microstructures evolved from the undulating grooves at lower $F_{\Sigma pulse}$ values, to bumpy structures, which further gave way to form the holes and the chaotic structures. At 1 kHz, the undulating grooves required a threshold $F_{\Sigma pulse}$ of 80 J/cm^2^. On increasing the $F_{\Sigma pulse}$ above 112 J/cm^2^, the bumpy structures started to appear, which with further increase in $F_{\Sigma pulse}$ gave way to form the holes and finally the chaotic microstructures. The holes required a threshold of 233 J/cm^2^ and the chaotic structures required a threshold of 400 J/cm^2^. However, at 10 kHz the threshold $F_{\Sigma pulse}$ values of the common microstructures were observed to have increased. For instance, the threshold $F_{\Sigma pulse}$ values of the undulating grooves increased to 90 J/cm^2^ at 10 kHz. Similarly, the threshold values of the other common microstructures, i.e., the bumpy structures, the holes, and the chaotic structures, increased to 165 J/cm^2^, 275 J/cm^2^, and 565 J/cm^2^, respectively, at 10 kHz, suggesting that an overall increase in the threshold $F_{\Sigma pulse}$ values is required for the microstructures to be reproducible at 10 kHz. This is unlike the case of Cu, where the threshold $F_{\Sigma pulse}$ values for the microstructures were observed to have decreased with an increase in the repetition rate.

### 3.3. Lacunarity Analysis

#### 3.3.1. Copper (Cu)

**Figure 5:**
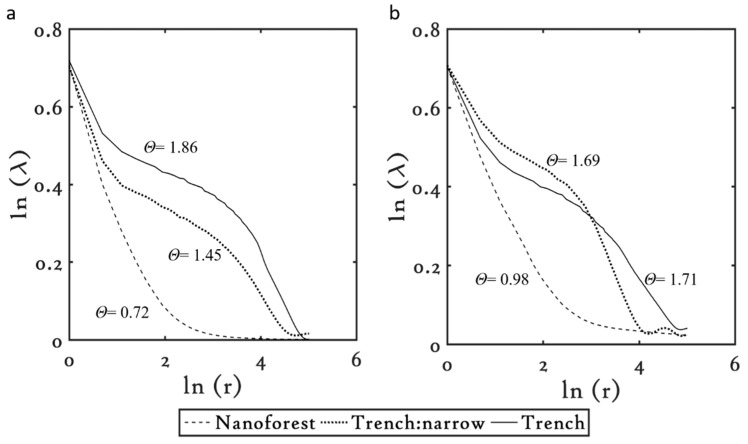
Lacunarity plots for the common microstructures on Cu at (**a**) 1 kHz and (**b**) 10 kHz.

For the purpose of comparison, only the lacunarity of the common microstructures were plotted. Figure 5 shows the lacunarity plots obtained from analyzing one SEM image per microstructure at 1 kHz and 10 kHz.

At 1 kHz, the spatial heterogeneity index, *θ*, was observed to increase as the microstructures evolved from nanoforest to the narrow trenches and finally to the deep trenches, having *θ* = 0.72, 1.45, and 1.86, respectively. Generally, an increase in the *θ* value indicates an increase in the structure coarseness. Consecutively, this indicates that the microstructures on Cu become more clumped or heterogeneous, i.e., lacked texture, as they evolved from nanoforest like structures to deep trenches. The coarseness of the deep trenches is higher than that for the narrow trenches, probably due to the thicker sections present in between the deep trenches as compared to the narrow trenches which adds to the spatial variation, and thus, makes them highly morphologically heterogeneous. At 10 kHz, similar trends in lacunarity were observed among the structures. Nanoforest structures are the least coarse, i.e., highly textured, having *θ* = 0.98, followed by the narrow trenches, having *θ* = 1.69, and finally followed by the deep trenches, which showed the maximum coarseness, i.e., least texture, with *θ* = 1.71.

The effect of repetition rate can be assessed by comparing the microstructures individually at the two repetition rates. As seen from the *θ* value, the nanoforest and the narrow trench-like structures appeared coarser when they were fabricated at 10 kHz rather than at 1 kHz. It is interesting to note the switchover in the coarseness of the microstructures as they evolve. The microstructures fabricated at lower $F_{\Sigma pulse}$ values, such as the nanoforest and the narrow trench-like structures (see Table 3), are coarser when micromachined at 10 kHz than at 1 kHz. As $F_{\Sigma pulse}$ is increased and the high $F_{\Sigma pulse}$ regime is reached, for instance where deep trenches were fabricated, the microstructures show higher coarseness when machined at 1 kHz in comparison to 10 kHz. Likewise, the results suggest that with an increase in the $F_{\Sigma pulse}$ values, the lacunarity of the microstructures at 10 kHz tends to reduce in comparison to their counterparts fabricated at 1 kHz. Although the exact reason behind such behavior observed in the microstructures at 10 kHz is still unclear, this behavior is further confirmed from the cross over seen in the lacunarity of the 10 kHz microstructures (Figure 5b). For instance, at 10 kHz, the deep trenches showed *θ* = 1.71 whereas the narrow trenches showed *θ* = 1.69 indicating that the deep trenches are coarser than the narrow trenches, yet we see the cross over indicating that the lacunarity of any structure machined at $F_{\Sigma pulse}$ values much higher than the deep trenches would show complete cross over.

#### 3.3.2. Titanium (Ti)

**Figure 6:**
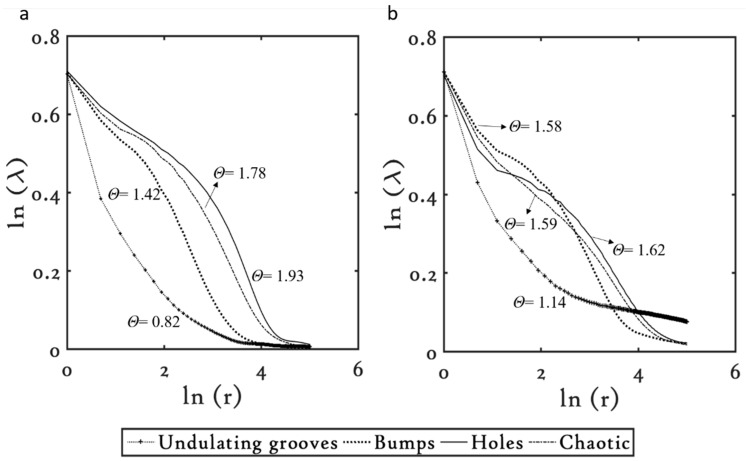
Lacunarity plots for the common microstructures on Ti at (**a**) 1 kHz and (**b**) 10 kHz.

The lacunarity plots of the common microstructures machined on Ti are shown in Figure 6. The spatial heterogeneity index, *θ*, of the microstructures at 1 kHz was observed to increase as they evolved with increasing $F_{\Sigma pulse}$. The undulating grooves that were fabricated at lower $F_{\Sigma pulse}$ were the least coarse structure with *θ* = 0.82, followed by the bumpy structures with *θ* = 1.42, followed by the holes with *θ* = 1.93. Interestingly, however, with further increase in the $F_{\Sigma pulse}$, which lead to the formation of the chaotic microstructures, the coarseness of the structures dropped. For instance, the chaotic structures showed *θ* = 1.78. The spatial heterogeneity of the chaotic structures was lower than the holes, probably because in chaotic structures the agglomerates that formed in the ridges in between tended to cover up the holes underneath, homogenizing the overall topology. Whereas, in the case of holes, the absence of the agglomerate structures adds on to the structural heterogeneity. Similar trends were observed in the lacunarity of the microstructures machined at 10 kHz.

As the microstructures evolved with increasing $F_{\Sigma pulse}$, their spatial heterogeneity increased, with undulating grooves being the least coarse, showing *θ* = 1.14, followed by the bumpy structures, showing *θ* = 1.58, and the holes, showing *θ* = 1.62. On further increasing $F_{\Sigma pulse}$, the coarseness of the microstructure reduced, as observed in the chaotic structures, showing *θ* = 1.59.

Comparison of the *θ* value for individual structures at the two repetition rates showed that the microstructures fabricated at lower $F_{\Sigma pulse}$ values are coarser when micromachined at 10 kHz. For instance, the undulating grooves and the bumpy microstructures that are machined at lower $F_{\Sigma pulse}$ values (see Table 3) appear coarser at 10 kHz.

As $F_{\Sigma pulse}$ is increased and the high $F_{\Sigma pulse}$ regime is reached, where for instance the holes and the chaotic microstructures were fabricated, the microstructures appear coarser when fabricated at 1 kHz. Alternatively, with an increase in the $F_{\Sigma pulse}$ values, the lacunarity of the microstructures at 10 kHz was observed to reduce in comparison to their counterparts fabricated at 1 kHz. Overall, the behavior in the lacunarity of the microstructures on Ti was very similar to what we observed on Cu; with increasing $F_{\Sigma pulse}$, the lacunarity of the microstructures at 10 kHz reduces and becomes lower than their counterparts at 1 kHz.

## 4. Discussion

It is interesting to see the different effects that pulse repetition rate has on the machining outcome. On Cu, the increase in repetition rate resulted in the lowering of the threshold $F_{\Sigma pulse}$ values of the microstructures, whereas for Ti, it resulted in an increase in the threshold $F_{\Sigma pulse}$ values. Ahmmed et al. [23], utilizing the material properties (*e-ph* coupling constant and thermal conductivity), provided a perception as to why different materials produce different microstructures under exactly alike machining conditions. Material properties along with laser-material interaction are the primary factors that govern the micromachining outcome. Additionally, secondary factors, such as melt dynamics or plasma formation [22], also play significant roles. To account for the differences observed on the machining outcome on the two materials with the change in repetition rate, we have considered the material properties and the prevailing material dynamics in between consecutive pulses.

It was suggested that the changes that occur in the material after the first pulse strikes largely decides the course of the consecutive pulse-material interaction [27]. In our experiment, the time interval between consecutive pulses at 1 kHz and 10 kHz is around 999 µs and 99 µs, respectively. During multiple femtosecond pulse ablation, a significant amount of thermal energy is deposited in metal samples causing localized heating which results in the formation of a melt layer [28,29,30,31]. If we consider the material properties, such as Cu that has a weak *e-ph* coupling constant (~0.48 × 10^17^ W/m^3^/K) [32] and high thermal conductivity (401 W/m/K), this combination of the material properties ensures quick dissipation of the thermal energy into the bulk, due to which the generated melt layer persists for only a short time. Ren et al. [33] modelled the lattice temperature variation of Cu and found that the melt layer induced by a single pulse of fluence 0.5 J/cm^2^ can persist roughly 200 ps, after which the resolidification process starts. During machining of Cu with overlapping pulses, as executed in our experiments, the pulse intervals at both 1 and 10 kHz are considerably long. The melt generated by one pulse resolidifies before the onset of the next pulse, leaving only structural defects on the material. Consequently, the consecutive laser pulses ablate on the resolidified surface. The structural defects on the material, resulting from the interaction of the material with the first pulse, and the resolidification process further enhance the absorption of the next incoming pulses [14,34,35]. At higher repetition rates, the number of defects generated in the material increases significantly due to a higher number of pulses per spot (PPS). This results in an increase in the energy absorption of the consecutive pulses, overall leading to the reduction in the ablation threshold. This is observed on Cu, where with an increase in repetition rate from 1 to 10 kHz, the ablation threshold reduces which is reflected in the reduction of the threshold $F_{\Sigma pulse}$ values of the microstructures (nanoforest: 120 J/cm^2^ (1 kHz), 88 J/cm^2^ (10 kHz); narrow trenches: 400 J/cm^2^ (1 kHz), 282 J/cm^2^ (10 kHz); deep trenches: 2101 J/cm^2^ (1 kHz), 353 J/cm^2^ (10 kHz)).

On the contrary, Ti has a strong *e-ph* coupling constant (~18.5 × 10^17^ W/m^3^/K) [32] and low thermal conductivity (~21.9 W/m/K), which ensures rapid energy transport from the electrons to the lattice but slow dissipation of the heat into the bulk, resulting in large amount of heat accumulation on the surface. Consequently, the melt layer persists for a longer time, approximately 300 µs [36]. If we consider the repetition rate, at 1 kHz the pulses are 999 µs apart. Therefore, while machining at 1 kHz, after the material has interacted with the first pulse, complete resolidification of the melt occurs before the arrival of the next pulse. Whereas at 10 kHz, the melt layer created by the first pulse persists when the next pulse hits, and it interacts with the melt rather than the resolidified material. Rapid irradiation of the melt causes the melt to proceed into a metastable state. As the temperature of the melt increases and reaches approximately 0.8 times the critical temperature (Tc), fluctuations observed in local density of the melt can lead to thermodynamic instability [37,38,39]. The onset of these fluctuations can cause rapid changes in the material properties, such as a loss of electrical conductivity due to many isolated regions of limited free electrons. It was shown that under these conditions, the optical transmission of the melt increases, its reflectivity decreases, and it acts as a dielectric. Likely this dielectric behavior of the melt is responsible for the increase in the ablation threshold at 10 kHz. For material ablation, free charge carriers are required that can oscillate in the electromagnetic field of the laser. Dielectrics, unlike metals, lack free charge carriers and therefore require additional energy, equivalent to the ionization potential, to generate the free charge carriers. Thus, in general, dielectrics have a higher ablation threshold than metals [40]. This higher ablation threshold is reflected in the higher threshold $F_{\Sigma pulse}$ values of the microstructures at 10 kHz (undulating grooves: 80 J/cm^2^ (1 kHz), 90 J/cm^2^ (10 kHz); bumps: 112 J/cm^2^ (1 kHz), 165 J/cm^2^ (10 kHz); holes: 233 J/cm^2^ (1 kHz), 275 J/cm^2^ (10 kHz); Chaotic: 400 J/cm^2^ (1 kHz), 565 J/cm^2^ (10 kHz)).

Additionally, with the change in repetition rate, we observed the appearance or disappearance of certain microstructures. For instance, the tree bark, stalagmite, fish scales, and aggregate structures on Cu and the aggregate and stalagmite structures on Ti were fabricated at 1 kHz but were not reported at 10 kHz. Although the precise reason behind such observation is still unclear, we believe that a preliminary explanation can be provided based on the melt dynamics. The formation mechanism of these microstructures depends on the competitive process between the nucleation and the melt dynamics, which is vastly decided by the experimental parameters. If noted carefully, the new microstructures on both Cu and Ti at 1 kHz (see Figure 3a and Figure 4a) appear over a narrow range of the $F_{\Sigma pulse}$ values, implying that a narrow range of experimental conditions is favorable for the production of these new microstructures. Tuning of the experimental parameters at the other repetition rate might not have led to the experimental conditions favorable for the production of these structures at that repetition rate.

Interestingly, in comparison to the opposing behavior observed in the effect of repetition rate on the threshold $F_{\Sigma pulse}$ values, the similar trends observed in the lacunarity of the microstructures on both the metals with changing repetition rate indicate that the changes in the surface texture with repetition rate is independent of the material properties. In general, with increasing $F_{\Sigma pulse}\;$we observed an increase in the spatial heterogeneity of the microstructures at both repetition rates. Nayak and Gupta [7] observed a decrease in the microstructure density with increasing fluence on Ti. The increase in spatial heterogeneity with increasing $F_{\Sigma pulse}$ in our microstructures could be attributed to the reduction in the microstructure density, resulting from more clumped structures at higher $F_{\Sigma pulse}$. However, the most important observation on the effect of the repetition rate on surface texture is the switch over in the microstructure lacunarity observed at higher $F_{\Sigma pulse}$, i.e., microstructures fabricated at lower $F_{\Sigma pulse}$ show higher lacunarity at 10 kHz, while at higher $F_{\Sigma pulse}$ the microstructures show higher lacunarity at 1 kHz. In this regard, it is important to note that for the structures where the switch over occurs, all have hierarchical structures; for instance, the holes and chaotic structures on Ti have agglomerates deposited on them, and they show higher spatial heterogeneity when micromachined at 1 kHz. Thus, the higher lacunarity seen in these structures at 1 kHz, compared to 10 kHz, is likely related to the presence of these agglomerates. The size of the agglomerates, formed due to vapor condensation [41], depends on the amount of vapor plume supersaturation, which in turn is determined by the plume temperature. Factors determining the plume temperature could be both the laser fluence as well as the heat accumulation resulting from the decrease in the pulse interval [42]. At higher $F_{\Sigma pulse}$ and at a higher repetition rate (10 kHz), the increase in the plume temperature decreases the plume supersaturation. It was suggested that the agglomerate size increases with the decrease in the supersaturation [43,44,45]. Most likely, the increase in the agglomerate size is responsible for the observed reduction in the spatial heterogeneity of the microstructures at 10 kHz. However, the trend observed in the lacunarity of the microstructures at lower $F_{\Sigma pulse}$ still remains unclear and needs to be considered in future studies.

## 5. Conclusions 

In conclusion, our study shows the effect of repetition rate on the micromachining outcome on metals. Cu and Ti were micromachined with <100 fs pulses at 1 kHz and 10 kHz repetition rates. Several homogenous microstructures were machined. While most of these microstructures were reproducible at both the repetition rates, certain microstructures were observed only at a specific repetition rate. The effect of repetition rate on structure formation was studied by comparing the threshold $F_{\Sigma pulse}$ values of the common microstructures at 1 kHz and 10 kHz and the effect of the repetition rate on surface texture was studied through lacunarity analysis. The microstructures on both metals showed similar trends in their lacunarity with changing repetition rate, i.e., microstructures fabricated at lower $F_{\Sigma pulse}$ values showed higher spatial heterogeneity at 10 kHz, whereas those fabricated at higher $F_{\Sigma pulse}$ values showed a higher spatial heterogeneity index at 1 kHz. However, the effect of changing the repetition rate on the threshold $F_{\Sigma pulse}$ were considerably different on both metals. On Cu, with the increase in repetition rate from 1 to 10 kHz, the threshold $F_{\Sigma pulse}$ values of the microstructures were observed to decrease, whereas on Ti, the threshold $F_{\Sigma pulse}$ values were observed to increase. These differences in the micromachining outcome were successfully addressed considering two important material properties (*e-ph* coupling constant and thermal conductivity). Alongside, our results show that the melt dynamics (such as the resolidification time) further contribute significantly to the micromachining outcome. Our study suggests that the micromachining outcome in any material at a particular repetition rate within the Hz–kHz range can be anticipated if these three material properties (*e-ph* coupling constant, thermal conductivity, and the resolidification time) are known.

## Figures and Tables

**Table 1:** Experimental conditions of various microstructures on metals.

Microstructures in Metals	Micromachining Parameters (Wavelength, Repetition Rate, Pulse Duration, Fluence)	Reference
Periodic nanostructures (LIPSS)		
Ni	800 nm, 1 kHz, 500 fs, 2.04 J/${\mathrm{cm}}^{2}$	Zuhlke et al. 2013 [19]
Al	800 nm, 100 Hz, 65 fs, 0.05 J/${\mathrm{cm}}^{2}$	Vorobyev and Guo 2008 [20]
Au	800 nm, 1 kHz, 65 fs, 0.16 J/${\mathrm{cm}}^{2}$	Vorobyev et al. 2007 [21]
Undulated groove microstructures		
Ti	800 nm, 1 kHz, 100 fs, 0.75 J/${\mathrm{cm}}^{2}$	Tsukamoto et al. 2006 [12]
Ni	not reported, 1 kHz, 50 fs, 1.392 J/${\mathrm{cm}}^{2}$	Zuhlke et al. 2013 [22]
Columnar microstructures		
Ti	800 nm, 1 kHz, 130 fs, 0.75 J/${\mathrm{cm}}^{2}$	Nayak and Gupta 2010 [7]
Ni	not reported, 1 kHz, 50 fs, 1.392–3.08 J/${\mathrm{cm}}^{2}$	Zuhlke et al. 2013 [22]
Al	800 nm, 1 kHz, 130 fs, <0.16 J/${\mathrm{cm}}^{2}$	Nayak and Gupta 2010 [7]
Bumpy microstructures		
Al	800 nm, 10 kHz, <100 fs, 0.4–1 J/${\mathrm{cm}}^{2}$	Ahmmed et al. 2015 [23]
Ti	800 nm, 10 kHz, <100 fs, 1.5–3 J/${\mathrm{cm}}^{2}$	Ahmmed et al. 2015 [23]
Conical microstructures		
Ti	800 nm, 1 kHz, 130 fs, 0.5–1.2 J/${\mathrm{cm}}^{2}$	Nayak and Gupta 2010 [7]
Ni	800 nm, 1 kHz, 50 fs, 0.12 J/${\mathrm{cm}}^{2}$	Zuhlke et al. 2013 [19]

**Table 2:** Microstructures fabricated at the two repetition rates.

Microstructures	Labels as in Figure 1 and Figure 2	1 kHz	10 kHz	Comment
**Cu**				
Nanoforest	Figure 1a	√	√	Common microstructures
Trench: narrow	Figure 1c	√	√	
Trench	Figure 1b	√	√	
Tree bark	Figure 1e	√	×	–
Stalagmite	Figure 1f	√	×	–
Fish scale	Figure 1g	√	×	–
Aggregate	Figure 1h	√	×	–
**Ti**				
Undulating grooves	Figure 2a	√	√	Common microstructures
Bumps	Figure 2b	√	√	
Holes	Figure 2c	√	√	
Chaotic	Figure 2d	√	√	
Trench	–	×	√	–
Stalagmite	Figure 2e	√	×	–
Aggregate	Figure 2f	√	×	–

**Table 3:** The threshold $F_{\Sigma pulse}$ values of the common microstructures at 1 and 10 kHz [23] (in J/cm^2^).

Common Microstructures	Labels as in Figure 1 and Figure 2	1 kHz	10 kHz
**Cu**			
Nanoforest	Figure 1a	120	88
Trench:narrow	Figure 1c	400	282
Trench	Figure 1b	2101	353
**Ti**			
Undulating grooves	Figure 2a	80	90
Bumps	Figure 2b	112	165
Holes	Figure 2c	233	275
Chaotic	Figure 2d	400	565

## Supplementary Materials

The following are available online at www.mdpi.com/1996-1944/9/12/1023/s1. Figure S1: Representative images of the microstructures on Cu machined at 10 kHz, Figure S2: Representative images of the microstructures on Ti machined at 10 kHz.
