# Supplementary material for: Effect of Repetition Rate on Femtosecond Laser-Induced Homogenous Microstructures
Source: Materials (Basel). 2016 Dec 19;9(12):1023. doi: 10.3390/ma9121023 (PMC5456961; doi:10.3390/ma9121023)
Supplement: Supplementary file 1 [file materials-09-01023-s001.pdf]

Supplementary Materials: Effect of Repetition Rate on Femtosecond Laser-Induced Homogenous Microstructures

Sanchari Biswas, Adya Karthikeyan and Anne-Marie Kietzig

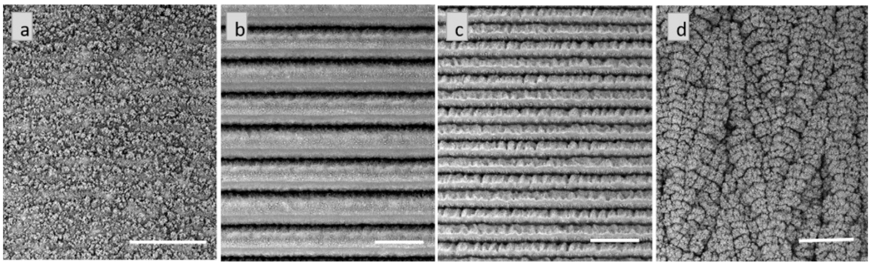

Figure S1. Representative images of the microstructures on Cu machined at 10 kHz. (**a**) Nanoforest; (**b**) Deep and well defined trenches; (**c**) Narrow trenches; (**d**) Rough and rugged chaotic structures. All scale bars represent 50 μm.

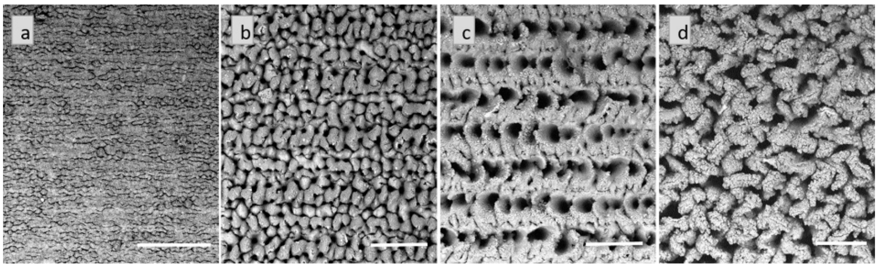

Figure S2. Representative images of the microstructures on Ti machined at 10 kHz. (**a**) Undulating grooves; (**b**) Bumpy structures; (**c**) Holes; (**d**) Chaotic structures. All scale bars represent 50 μm.
